# Biomarkers and clinical scores to aid the identification of disease severity and intensive care requirement following activation of an in-hospital sepsis code

**DOI:** 10.1186/s13613-020-0625-5

**Published:** 2020-01-15

**Authors:** Jaume Baldirà, Juan Carlos Ruiz-Rodríguez, Darius Cameron Wilson, Adolf Ruiz-Sanmartin, Alejandro Cortes, Luis Chiscano, Roser Ferrer-Costa, Inma Comas, Nieves Larrosa, Anna Fàbrega, Juan José González-López, Ricard Ferrer

**Affiliations:** 1 Intensive Care Department, Hospital de la Santa Creu i Sant Pau, Barcelona, Spain; 2 Department de Medicina, Universitat Autònoma de Barcelona, Barcelona, Spain; 3 Intensive Care Department, Vall d’Hebron University Hospital, Barcelona, Spain; 4 Shock, Organ Dysfunction and Resuscitation Research Group, Vall d’Hebron Institute of Research, Barcelona, Spain; 5 Biochemistry Department, Clinical Laboratories, Vall d’Hebron University Hospital, Barcelona, Spain; 6 Microbiology Department, Vall d’Hebron University Hospital, Barcelona, Spain; 7 Department de Genètica i Microbiologia, Universitat Autònoma de Barcelona, Barcelona, Spain

## Abstract

**Background:**

Few validated biomarker or clinical score combinations exist which can discriminate between cases of infection and other non-infectious conditions following activation of an in-hospital sepsis code, as well as provide an accurate severity assessment of the corresponding host response. This study aimed to identify suitable blood biomarker (MR-proADM, PCT, CRP and lactate) or clinical score (SOFA and APACHE II) combinations to address this unmet clinical need.

**Methods:**

A prospective, observational study of patients activating the Vall d’Hebron University Hospital sepsis code (ISC) within the emergency department (ED), hospital wards and intensive care unit (ICU). Area under the receiver operating characteristic (AUROC) curves, logistic and Cox regression analysis were used to assess performance.

**Results:**

148 patients fulfilled the Vall d’Hebron ISC criteria, of which 130 (87.8%) were retrospectively found to have a confirmed diagnosis of infection. Both PCT and MR-proADM had a moderate-to-high performance in discriminating between infected and non-infected patients following ISC activation, although the optimal PCT cut-off varied significantly across departments. Similarly, MR-proADM and SOFA performed well in predicting 28- and 90-day mortality within the total infected patient population, as well as within patients presenting with a community-acquired infection or following a medical emergency or prior surgical procedure. Importantly, MR-proADM also showed a high association with the requirement for ICU admission after ED presentation [OR (95% CI) 8.18 (1.75–28.33)] or during treatment on the ward [OR (95% CI) 3.64 (1.43–9.29)], although the predictive performance of all biomarkers and clinical scores diminished between both settings.

**Conclusions:**

Results suggest that the individual use of PCT and MR-proADM might help to accurately identify patients with infection and assess the overall severity of the host response, respectively. In addition, the use of MR-proADM could accurately identify patients requiring admission onto the ICU, irrespective of whether patients presented to the ED or were undergoing treatment on the ward. Initial measurement of both biomarkers might therefore facilitate early treatment strategies following activation of an in-hospital sepsis code.

## Introduction

An early diagnosis of sepsis, irrespective of hospitalisation setting, is crucial in order to provide a rapid and appropriate therapeutic response. This, however, may be complicated by the complex and heterogeneous host response to infection, as well as the high level of patient heterogeneity between departments [1].

A standardised set of defined criteria to aid in the early identification of sepsis therefore remains elusive, despite the established use of specific parameters in conditions such as myocardial infarction and pulmonary embolism [2]. Furthermore, the poor acceptance of previous [3] and current [4, 5] definitions has led to significant debate concerning the most appropriate clinical criteria required to make an accurate diagnosis. Differential presenting signs, symptoms and severities at each stage of hospitalisation, as well as prior surgical history and the setting of the original infectious insult, significantly exacerbate this already complex diagnosis.

The construction and validation of new parameters to aid in the early diagnosis and severity assessment of sepsis patients is therefore crucial. The recent development of tools such as the quick Sequential Organ Failure Assessment (qSOFA) score to screen patients at risk of a poor outcome has resulted in additional parameters becoming available to the treating clinician [6]; however, significant inherent and kinetical limitations make its implementation problematic [7–9]. The use of a panel of blood biomarkers, however, may provide a more elegant solution. Whilst measurement of individual biomarkers to concurrently discriminate between infectious and non-infectious patients and provide an accurate assessment of disease severity is highly desirable [10], no such biomarker appears to have a high enough accuracy to fulfil both clinical requirements. Accordingly, both requirements should be considered separately.

The use of procalcitonin (PCT) and C-reactive protein (CRP) to discriminate between infectious and non-infectious cases is well documented [11–15], despite neither biomarker being well-established in the field of emergency medicine or on the medical ward. Furthermore, neither biomarkers exhibit a robust performance in terms of mortality prediction or in the identification of patients requiring intensive care (ICU) admission. Conversely, the novel blood biomarker, mid-regional proadrenomedullin (MR-proADM) has been shown to be elevated in the early stages of infectious disease progression in the emergency department (ED) [16], after severe burn injury on a medical ward [17], and in critically ill sepsis patients with decreasing PCT concentrations [1] who may require renal replacement therapy (RRT) [18], or are likely to progress towards multiple organ failure [19].

This study therefore aimed to investigate the performance of each biomarker (MR-proADM, PCT, CRP and lactate) and clinical score (SOFA and APACHE II) in patients fulfilling the criteria for the Vall d’Hebron University in-hospital sepsis code (ISC) in order to: (1) discriminate between infected and non-infected patients; (2) assess infection severity according to 28- and 90-day mortality prediction, and (3) identify patients presenting to the ED or undergoing treatment on a medical/surgical ward who require subsequent ICU admission.

## Methods

### Study design and ethical approval

This single-centre, observational study prospectively enrolled consecutive patients across all hospital departments (ED, ward and ICU) who met the criteria for the activation of the Vall d’Hebron University Hospital in-hospital sepsis code (ISC) [20] between November 2016 and August 2017. The study protocol was approved by the Clinical Research Ethics Committee of Vall d’Hebron University Hospital (PR(AG)333/2016) without the need for informed consent, and was performed in accordance with the ethical standards laid down in the 1964 Declaration of Helsinki and its later amendments.

### Inclusion and exclusion criteria

Inclusion criteria comprised adult patients ≥ 18 years of age presenting with either a suspected or documented infection with the presence of at least one of the two following sets of variables, as outlined by the Vall d’Hebron University Hospital in-hospital sepsis code (ISC) [20]: (1) an acute alteration in the level of consciousness not explained by other clinical conditions, or (2) the presence of hyperthermia (axillary temperature > 38.3 °C) or hypothermia (axillary temperature < 36.0 °C), and/or tachycardia (> 110 beats per minute), tachypnea (> 30 breaths per minute) or desaturation (SpO_2_ < 90%), as well as arterial hypotension (systolic arterial pressure < 90 mmHg or mean arterial pressure < 65 mmHg, or a decrease of > 40 mmHg of baseline systolic arterial pressure). Exclusion criteria included non-adult patients, pregnancy, or patients where no blood sample could be obtained.

### Study endpoints and analytical aims

Study endpoints and analytical aims were defined as follows. *Infection identification*: either proven infection resulting from a positive blood culture or microbial identification, or a final clinical diagnosis of infection. *28*- *and 90*-*day mortality*: all-cause mortality within either 28 or 90 days following enrolment. *Community-acquired infection*: infectious insult developed outside the hospital within the community. *Hospital-acquired infection*: infectious insult developed whilst a patient was hospitalised for a previously non-infectious complaint. *ICU admission*: admission onto either a medical or surgical intensive care unit within 28 days of sepsis code activation.

### Data collection and biomarker measurements

Patient comorbidities and demographics were subsequently noted upon sepsis code activation, as well as data concerning triage, routine laboratory values, microbiology testing and final clinical diagnosis. Clinical scores (APACHE II and SOFA) were retrospectively calculated whenever possible upon enrolment. The following biomarker measurements were performed as standard: PCT using a chemiluminescent immunoassay (CLIA), CRP using an immuno-turbidimetric test, and L-lactate using an enzymatic colour test. MR-proADM was retrospectively analysed following blood sampling through a central catheter, and stored at − 80 °C until batch analysis using TRACE technology (time-resolved amplified cryptate emission, KRYPTOR platform, Thermo Fisher). Results were unavailable to the treatment physician throughout patient enrolment and hospitalisation.

### Statistical analysis

Data were either reported using the mean (standard deviation) for symmetrically distributed variables, or the median [first quartile − third quartile] for variables showing a skewed distribution. Demographic and clinical characteristics between surviving and non-surviving patients up to 28 days following sepsis code activation were assessed using the Chi-square (*χ*^2^) test for categorical variables, Student’s *t* test for age, and the Mann–Whitney *U* test for all other continuous variables. Area under the receiver operating characteristic (AUROC) curves were used to identify the biomarker or clinical score with the greatest predictive value for each endpoint, with 95% confidence intervals (95% CI) compared to determine significance. Youden’s criterion established optimal cut-off values with corresponding sensitivity and specificity values. Univariate and multivariate Cox regression models assessed the association with survival time (censored at 28 days following sepsis code activation), with potential confounding variables selected based on a univariate analysis (*p*-value < 0.005 after applying a Bonferroni correction), and subsequently included in a multivariate analysis. Results were either presented as the hazard (HR) or odds ratio (OR) per 1 interquartile-range increase for mortality prediction and ICU admission, respectively, with corresponding 95% confidence intervals. Previously established [16] biomarker cut-offs for 28-day mortality prediction were further analysed within this dataset, and mortality rates in patient subgroups with high biomarker values compared to those with low values. All data were analysed using the statistics software R (version 3.1.2).

## Results

### Patient characteristics

A total of 150 patients were identified and enrolled after activation of the sepsis code (sepsis code group), comprising 43 (29.1%) presenting to the emergency department (ED), 70 (47.3%) already undergoing treatment on a medical or surgical ward, and 35 (23.6%) already present on the intensive care unit (ICU). 2 patients were removed from the analysis due to a lack of blood sample.

### Infection identification following sepsis code activation

**Fig. 1:**
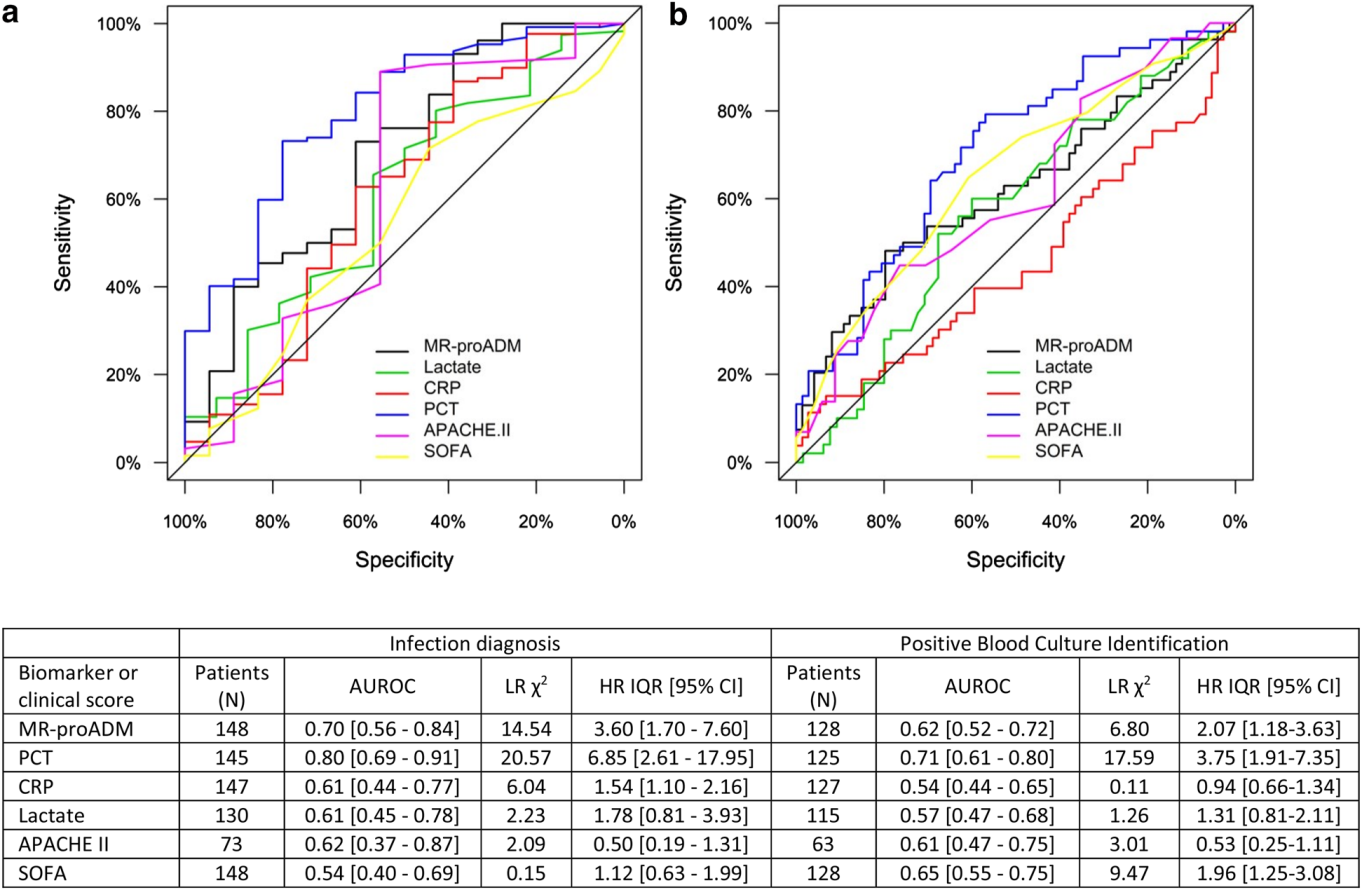
AUROC and univariate analysis for **a** discriminating between patients with a final diagnosis of infection from those where no infection was found, after activation of an in-hospital sepsis code, and **b** prediction of a positive blood culture in patients with a final diagnosis of infection. *APACHE II* Acute Physiological and Chronic Health Evaluation II score, *AUROC* area under the receiver operating characteristic curve, *CI* confidence interval, *CRP* C-reactive protein, *HR* hazard ratio, *IQR* interquartile range, *LR* likelihood ratio, *MR*-*proADM*: mid-regional proadrenomedullin, *N* number, *PCT* procalcitonin, *SOFA* Sequential Organ Failure Assessment score

Based on final clinical diagnosis, a total of 130 (87.8%) infected (sepsis code infected group) and 18 (12.2%) non-infected patients (sepsis code non-infected group) could be retrospectively identified following initial sepsis code activation. Patient characteristics of the infected population are presented in Table 1. PCT was found to have a high discriminatory performance (Fig. 1a), with an optimal cut-off of 2.02 ng/mL (sensitivity and specificity: 0.73 and 0.78). This cut-off, however, varied considerably depending on the location of sepsis code activation, with an optimal cut-off of 0.37 ng/mL (sensitivity and specificity: 0.86 and 0.75) in the ED, 1.91 ng/mL (sensitivity and specificity: 0.77 and 0.86) on the ward and 2.02 mg/mL (sensitivity and specificity: 0.79 and 0.71) on the ICU.

**Table 1:** Clinical patient characteristics upon activation of the sepsis code with respect to the total infected patient population and subsequent 28-day mortality

	Patient population (*N* = 130)	Survivors (*N* = 102)	Non-survivors (*N* = 28)	*p*-value
Age (years) (mean, S.D.)	63.1 (15.2)	62.3 (15.2)	66.1 (15.0)	0.205
Male gender (*N*, %)	83 (63.8%)	61 (59.8%)	22 (78.6%)	0.348
Infection characterisation				
Infection no SIRS (*N*, %)	7 (5.4%)	6 (5.9%)	1 (3.6%)	0.623
Severe sepsis (*N*, %)	41 (31.8%)	37 (36.3%)	4 (14.3%)	0.028
Septic shock (*N*, %)	82 (63.1%)	59 (57.8%)	23 (82.1%)	0.031
Sepsis-2 (*N*, %)	122 (93.8%)	95 (93.1%)	27 (96.4%)	0.814
Sepsis-3 (*N*, %)	116 (89.2%)	88 (86.3%)	28 (100.0%)	0.157
Location of sepsis code activation				
Emergency department (*N*, %)	39 (30.0%)	33 (32.4%)	6 (21.4%)	0.568
Ward (*N*, %)	63 (48.5%)	47 (46.1%)	16 (57.1%)	0.729
ICU (*N*, %)	28 (21.5%)	22 (21.6%)	6 (21.4)	0.839
Surgical admissions (*N*, %)	47 (36.2%)	37 (36.3%)	10 (35.7%)	0.487
Medical admissions (*N*, %)	83 (63.8%)	65 (63.7%)	18 (64.3%)	0.487
ICU length of stay (days) (median, IQR)	6 [2–15]	5 [2–11]	12 [3–24]	0.093
Hospital length of stay (days) (median, IQR)	16.5 [8–31]	17 [8–33]	16 [5.5–27]	0.298
Life supporting and intensive care therapies				
Vasopressors (*N*, %)	67 (51.5%)	52 (40.0%)	15 (53.6%)	0.106
Renal replacement therapy (*N*, %)	15 (11.5%)	8 (6.2%)	7 (25.0%)	0.009
Mechanical ventilation (*N*, %)	44 (33.8%)	30 (23.1%)	14 (50.0%)	0.011
Mechanical ventilation duration (days)	7 [3–16.25]	5 [3–17.5]	10 [4.5–14.75]	0.081
High-flow nasal cannula use (*N*, %)	19 (14.6%)	16 (15.7%)	3 (10.7%)	0.386
Pre-existing comorbidities				
Cardiopathy (*N*, %)	24 (18.5%)	16 (15.7%)	8 (28.6%)	0.120
Chronic kidney disease (*N*, %)	19 (14.6%)	14 (13.7%)	5 (17.6%)	0.584
COPD (*N*, %)	19 (14.6%)	10 (9.8%)	9 (32.1%)	0.003
Immunosuppression (*N*, %)	63 (48.5%)	44 (43.1%)	19 (67.9%)	0.020
Liver cirrhosis (*N*, %)	3 (2.3%)	1 (1.0%)	2 (7.1%)	0.054
Microbiology				
Positive blood culture (*N*, %)	54 (41.5%)	40 (39.2%)	14 (50.0%)	0.238
Gram positive (*N*, %)	34 (26.2%)	24 (23.5%)	10 (35.7%)	0.895
Gram negative (*N*, %)	17 (13.1%)	13 (12.7%)	4 (14.3%)	0.797
Fungal (*N*, %)	2 (1.5%)	1 (1.0%)	2 (7.1%)	0.529
Origin of infection				
Abdominal (*N*, %)	41 (31.5%)	35 (34.3%)	6 (21.4%)	0.194
Bacteria—primary (*N*, %)	4 (3.1%)	3 (2.9%)	1 (3.6%)	0.864
Catheter-related (*N*, %)	8 (6.2%)	5 (4.9%)	3 (10.7%)	0.257
Central nervous system (*N*, %)	2 (1.5%)	1 (1.0%)	1 (3.6%)	0.324
Respiratory (*N*, %)	34 (26.2%)	25 (24.5%)	9 (32.1%)	0.416
Soft-tissue (*N*, %)	4 (3.1%)	4 (3.9%)	0 (0.0%)	0.287
Urinary (*N*, %)	30 (23.1%)	25 (24.5%)	5 (17.9%)	0.459
Unknown (*N*, %)	3 (2.3%)	1 (1.0%)	2 (7.1%)	0.054
Other (*N*, %)	4 (3.1%)	3 (2.9%)	1 (3.6%)	0.864
Source control				
Focus cleaning (*N*, %)	34 (26.2%)	28 (27.5%)	6 (21.4%)	0.521
Drainage (*N*, %)	8 (6.2%)	6 (5.9%)	2 (7.1%)	0.806
Surgery (*N*, %)	23 (17.7%)	20 (19.6%)	3 (10.7%)	0.275
Biomarker and severity scores				
MR-proADM (nmol/L) (median, IQR)	3.54 [1.89–6.69]	3.18 [1.73–5.64]	5.69 [3.98–13.43]	< 0.001
PCT (ng/mL) (median, IQR)	7.05 [1.46–28.8]	7.85 [1.61–29.13]	4.94 [1.25–29.86]	0.654
Lactate (mmol/L) (median, IQR)	2.65 [1.70–4.57]	2.50 [1.60–3.75]	4.1 [2.0–8.10]	0.005
CRP (mg/L) (median, IQR)	17.78 [11.03–27.88]	17.48 [10.83–27.60]	23.14 [12.86–30.80]	0.322
SOFA (points) (mean, S.D.)	6.39 (3.46)	5.74 (3.11)	8.65 (3.70)	< 0.001
APACHE II (points) (mean, S.D.)	21.98 (7.22)	21.63 (7.58)	23.13 (6.02)	0.783

Data are presented as absolute numbers with percentages in brackets, indicating the proportion of surviving and non-surviving patients at 28 days. *APACHE II* Acute Physiological and Chronic Health Evaluation II score, *COPD* chronic obstructive pulmonary disease, *CRP* C-reactive protein, *ICU* intensive care unit, *IQR* interquartile range, *MR*-*proADM* mid-regional proadrenomedullin, *N* number, *PCT* procalcitonin, *SOFA* Sequential Organ Failure Assessment score

In the sepsis code infected group, a positive blood culture rate was obtained in 54 (42.2%) cases, with a greater prevalence of Gram-positive as opposed to Gram-negative infections (*N *= 34; 26.2% vs. *N *= 17; 13.1%, *p *< 0.01). A comparison of biomarker and clinical score performance found that PCT had a moderate performance in predicting the likelihood of a positive culture (Fig. 1b). The addition of further biomarkers or clinical scores to initial PCT measurements resulted in no significant increase in discriminatory (infected vs. non-infected patients) or predictive (positive vs. negative blood culture) performance.

## 28- and 90-day mortality prediction

The 28-day all-cause mortality rate within the sepsis code infected group was 21.5% (*N *= 28), with a higher mortality rate in patients enrolled whilst on the ward (*N *= 16; 25.4%) and ICU (*N *= 6; 21.4%) as opposed to in the ED (*N *= 6; 15.4%; Table 1). The 90-day all-cause mortality rate was 24.6% (*N *= 32). In both cases, MR-proADM, lactate and SOFA were all significantly elevated in non-surviving as opposed to surviving patients (*p *< 0.01), whereas no significant differences were found in PCT, CRP or APACHE II values.

**Fig. 2:**
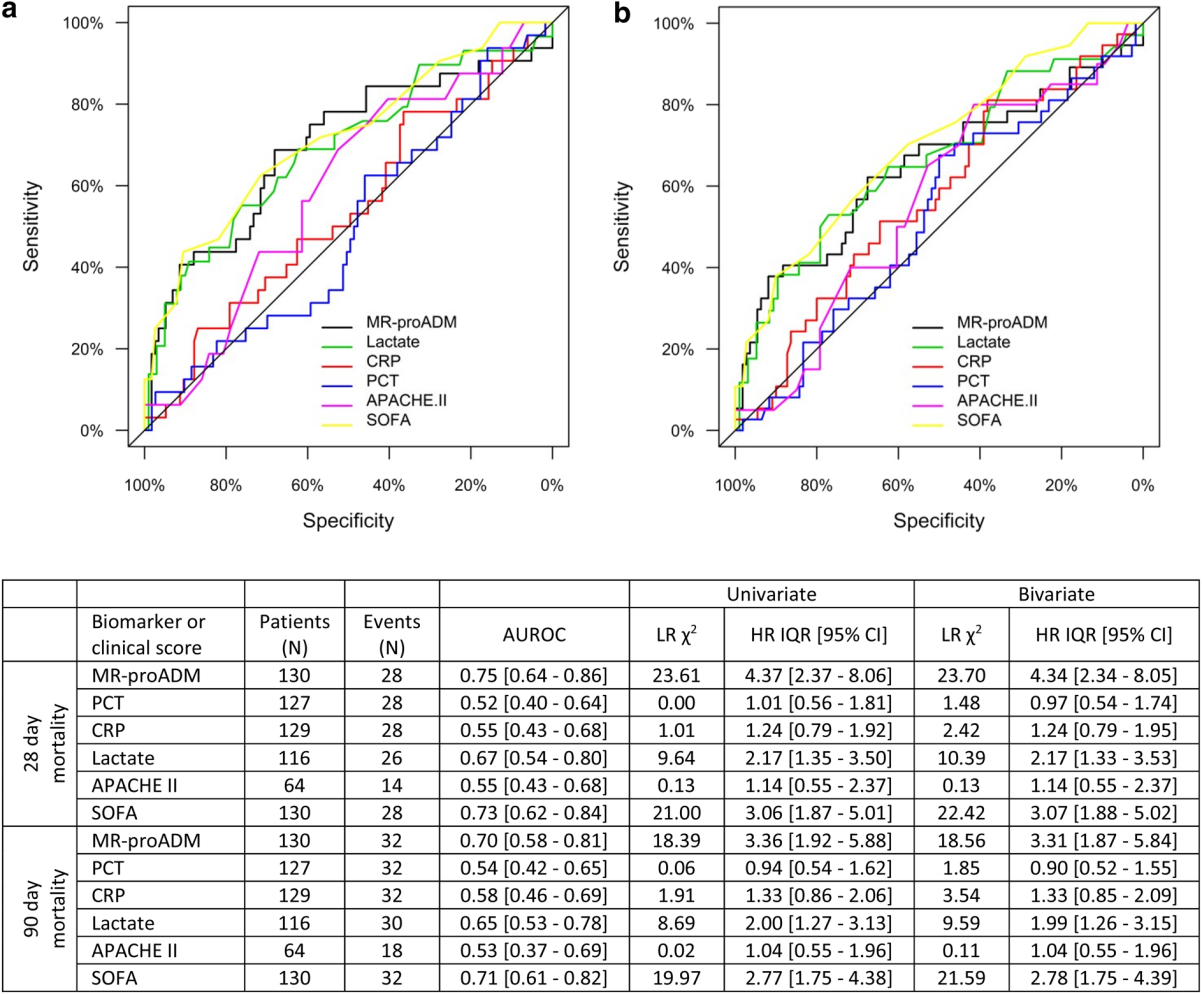
AUROC, univariate and bivariate analysis for the prediction of 28-day **a** and 90-day **b** mortality for each biomarker and clinical score. *APACHE II* Acute Physiological and Chronic Health Evaluation II score, *AUROC* area under the receiver operating characteristic curve, *CI* confidence interval, *CRP* C-reactive protein, *HR* hazard ratio, *IQR* interquartile range, *LR* likelihood ratio, *MR*-*proADM* mid-regional proadrenomedullin, *N* number, *PCT* procalcitonin, *SOFA* Sequential Organ Failure Assessment score

AUROC analysis found that MR-proADM performed well in predicting 28-day mortality, followed by SOFA and lactate, with an optimal cut-off of 4.28 nmol/L (sensitivity and specificity: 0.76 and 0.65). No significant association was observed with either PCT, CRP or APACHE II (Fig. 2). Similar findings were observed in both the univariate Cox regression analysis and after adjusting for the influence of existing chronic obstructive pulmonary disease (COPD), which was the only pre-existing variable found to significantly influence mortality prediction. The addition of PCT to MR-proADM measurements further improved predictive performance [HR (95% CI) 7.5 (3.7–15.2)], greater than any other biomarker or clinical score combination. Similar results were also obtained for 90-day mortality prediction, with a moderate performance found using MR-proADM, followed by SOFA and lactate. The influence of existing COPD had little impact on predictive performance, whilst the addition of PCT to MR-proADM resulted in the greatest increase in predictive performance [HR (95% CI) 5.7 (3.0–10.9)]. Interestingly, no increase in performance was found for the combination of PCT with either SOFA or lactate measurements [HR (95% CI) 2.9 (1.9–4.6) and 2.1 (1.3–3.2)], respectively).

Using an MR-proADM cut-off of 1.54 nmol/L within the infected patient population, 108 (83.1%) patients were found to have values equal to or exceeding this cut-off, with a corresponding mortality rate of 24.1% (*N *= 26). In comparison, patients with MR-proADM values < 1.54 nmol/L (*N *= 22, 16.9%) had a significantly (*p *< 0.01) lower mortality rate of 13.6% (*N *= 3). No patient died with an MR-proADN value of < 0.88 nmol/L. Only 6 (4.6%) patients were found to have PCT concentrations < 0.25 μg/mL, of which one died within 28 days.

### Mortality prediction based on location of infection development and reason for patient admission

Subgroup analysis found that a total of 67 (51.5%) patients developed an infection in the community and were subsequently hospitalised, compared to 48 (36.9%) patients where the infection was acquired during hospitalisation despite being admitted for a non-infectious related condition. The 28-day mortality rates of both groups were, however, similar (*N *= 15; 22.4% vs. *N *= 12; 25.0%). Likewise, 47 (36.2%) patients were identified using the sepsis code following a surgical procedure, as opposed to 83 (63.8%) patients presenting with a medical complaint, again with similar 28-day mortality rates between groups (*N *= 10; 21.3% vs. *N *= 18; 21.7%).

AUROC and Cox regression analysis found that MR-proADM had a high predictive value in patients with community-acquired infections, followed by SOFA and lactate (Table 2), which could be further increased after the addition of PCT [HR (95% CI) 10.0 (3.6–27.5)]. The optimal MR-proADM cut-off was 3.67 nmol/L, with a sensitivity and specificity of 0.93 and 0.52, respectively. Conversely, the SOFA score had high predictive performance in patients with hospital-acquired infections (Table 2). Interestingly, the combination of MR-proADM with APACHE II resulted in the largest increase in predictive value [HR (95% CI) 7.9 (1.5–41.1)], whereas the addition of PCT to MR-proADM only resulted in a small overall additive effect. Similarly, AUROC and Cox regression analysis found that MR-proADM had a moderate-to-high predictive value within both medical and surgical patient subgroups, followed by SOFA and lactate (Table 3). The combination of SOFA and APACHE II scores resulted in the greatest predictive performance in the surgical cohort [HR (95% CI) 9.36 (2.13–4.17)], compared to the combination of MR-proADM and PCT in the medical cohort [HR (95% CI) 9.30 (3.89–22.25)].

**Table 2:** 28-day mortality prediction in patients with either community or hospital-acquired infections

	Biomarker or clinical score	Patients (N)	Events (N)	AUROC	Univariate		Bivariate	
					LR *χ*^2^	HR IQR [95% CI]	LR *χ*^2^	HR IQR [95% CI]
Community	MR-proADM	67	15	0.76 [0.64–0.89]	13.08	4.60 [1.95–10.83]	14.52	4.88 [1.96–12.14]
	PCT	65	15	0.54 [0.36–0.71]	0.09	0.89 [0.43–1.85]	1.64	0.89 [0.43–1.85]
	CRP	66	15	0.57 [0.40–0.74]	0.48	1.28 [0.62–2.63]	2.30	1.32 [0.62–2.80]
	Lactate	60	14	0.71 [0.53–0.89]	10.03	3.44 [1.59–7.45]	10.61	3.09 [1.40–6.83]
	APACHE II	33	8	0.70 [0.50–0.90]	2.10	2.07 [0.77–5.55]	2.79	2.14 [0.76–6.03]
	SOFA	67	15	0.73 [0.57–0.88]	8.93	2.85 [1.39–5.85]	11.16	3.01 [1.44–6.28]
Hospital	MR-proADM	48	11	0.66 [0.45–0.88]	5.59	3.15 [1.19–8.37]	5.70	3.32 [1.19–9.25]
	PCT	48	11	0.46 [0.27–0.65]	0.03	0.91 [0.30–2.77]	0.19	0.88 [0.29–2.70]
	CRP	48	11	0.53 [0.32–0.73]	0.52	1.20 [0.70–2.07]	0.61	1.19 [0.69–2.06]
	Lactate	43	10	0.58 [0.35–0.81]	0.87	1.40 [0.70–2.77]	0.87	1.40 [0.70–2.77]
	APACHE II	25	6	0.63 [0.37–0.89]	0.85	0.61 [0.21–1.74]	1.18	0.58 [0.19–1.75]
	SOFA	48	11	0.78 [0.61–0.95]	12.12	3.60 [1.70–7.59]	12.18	3.58 [1.70–7.55]

*APACHE II* Acute Physiological and Chronic Health Evaluation II score, *AUROC* area under the receiver operating characteristic curve, *CI* confidence interval, *CRP* C-reactive protein, *HR* hazard ratio, *IQR* interquartile range, *LR* likelihood ratio of the *χ*^2^ test, *MR*-*proADM* mid-regional proadrenomedullin, *N* number, *PCT* procalcitonin, *SOFA* Sequential Organ Failure Assessment score

**Table 3:** 28-day mortality prediction in patients enrolled following either surgical or medical complaints

	Biomarker or clinical score	Patients (N)	Events (N)	AUROC	Univariate		Bivariate	
					LR *χ*^2^	HR IQR [95% CI]	LR *χ*^2^	HR IQR [95% CI]
Surgical	MR-proADM	47	10	0.80 [0.66–0.94]	9.03	6.53 [1.65–25.88]	10.40	6.34 [1.84–41.04]
	PCT	47	10	0.47 [0.26–0.68]	0.34	1.39 [0.46–4.21]	0.68	1.56 [0.48–5.10]
	CRP	47	10	0.59 [0.38–0.79]	0.37	1.24 [0.60–2.58]	0.55	1.26 [0.60–2.63]
	Lactate	44	8	0.73 [0.49–0.97]	3.97	2.72 [1.00–7.39]	4.06	2.65 [0.98–7.20]
	APACHE II	29	7	0.53 [0.29–0.77]	0.01	1.05 [0.39–2.80]	0.78	0.86 [0.29–2.53]
	SOFA	47	10	0.74 [0.56–0.92]	10.86	5.05 [1.82–14.02]	11.12	5.55 [1.87–16.46]
Medical	MR-proADM	83	18	0.72 [0.57–0.87]	15.05	3.84 [1.96–7.54]	16.32	3.87 [1.90–7.86]
	PCT	80	18	0.55 [0.40–0.71]	0.10	0.89 [0.44–1.79[	2.89	0.90 [0.45–1.80]
	CRP	82	18	0.46 [0.30–0.61]	0.63	1.23 [0.71–2.15]	3.49	1.27 [0.70–2.29]
	Lactate	72	18	0.66 [0.51–0.82]	6.43	2.06 [1.21–3.51]	7.84	1.99 [1.14–3.46]
	APACHE II	35	7	0.59 [0.34–0.82]	0.20	1.28 [0.44–1.79]	0.43	1.17 [0.38–3.59]
	SOFA	83	18	0.71 [0.57–0.86]	16.32	3.87 [1.90–7.86]	13.33	2.50 [1.43–4.36]

*APACHE II* Acute Physiological and Chronic Health Evaluation II score, *AUROC* area under the receiver operating characteristic curve, *CI* confidence interval, *CRP* C-reactive protein, *HR* hazard ratio, *IQR* interquartile range, *LR* likelihood ratio of the *χ*^2^ test, *MR*-*proADM* mid-regional proadrenomedullin, *N* number, *PCT* procalcitonin, *SOFA* Sequential Organ Failure Assessment score

### Intensive care unit admission after sepsis code activation

Finally, biomarker and clinical score performance was compared in order to predict admission to both the surgical and medical intensive care units. Activation of the sepsis code in both the ED and wards resulted in a high rate of admission on the same or subsequent day, with a total of 16 (41.0%) patients transferred from the ED and 28 (45.9%) patients transferred from the ward. MR-proADM showed a moderate-to-high predictive value in both settings, with similar results also found after univariate logistic regression (Table 4).

**Table 4:** Intensive (ICU) and surgical intensive (ICU) care unit admission from the emergency department and ward

	Biomarker or clinical score	Patients (N)	Events (N)	AUROC	Univariate	
					LR *χ*^2^	OR IQR [95% CI]
Emergency department	MR-proADM	39	16	0.80 [0.66–0.94]	10.55	8.18 [1.75–28.33]
	PCT	37	16	0.74 [0.57–0.90]	7.18	3.59 [1.27–10.12]
	CRP	38	16	0.64 [0.46–0.82]	2.83	1.98 [0.85–4.60]
	Lactate	33	14	0.45 [0.24–0.65]	0.72	0.60 [0.18–1.99]
	APACHE II	16	14	0.63 [0.33–0.92]	0.21	1.63 [0.20–13.47]
	SOFA	39	16	0.78 [0.64–0.93]	10.21	5.04 [1.59–15.99]
Ward admission	MR-proADM	63	28	0.72 [0.64–0.79]	10.1	3.64 [1.43–9.29]
	PCT	63	28	0.59 [0.46–0.72]	3.5	1.85 [0.93–3.69]
	CRP	63	28	0.57 [0.41–0.65]	0.8	0.76 [0.41–1.41]
	Lactate	63	28	0.57 [0.44–0.70]	1.7	1.49 [0.81–2.74]
	APACHE II	20	16	0.69 [0.40–0.77]	1.3	0.38 [0.06–2.27]
	SOFA	63	28	0.64 [0.49–0.75]	3.8	2.03 [0.97–4.24]

*APACHE II* Acute Physiological and Chronic Health Evaluation II score, *AUROC* area under the receiver operating characteristic curve, *CI* confidence interval, *CRP* C-reactive protein, *IQR* interquartile range, *LR* likelihood ratio of the *χ*^2^ test, *MR*-*proADM* mid-regional proadrenomedullin, *N* number, *OR* odds ratio, *PCT* procalcitonin, *SOFA* Sequential Organ Failure Assessment score

## Discussion

This study compared the performance of a number of clinical severity scores, conventional and novel biomarkers following activation of an in-hospital sepsis code, in order to identify cases of infection and assess the severity of corresponding host response. Results found that the use of PCT to discriminate between sepsis code infected group and sepsis code non-infected group, and MR-proADM to identify disease severity irrespective of the location of infectious insult, had the greatest accuracy in fulfilling both criteria. Interestingly, MR-proADM was also the most accurate parameter in identifying patients presenting to the ED or receiving treatment on a medical ward who subsequently required immediate ICU admission.

The use of clinical or laboratory parameters which are significantly increased in the early stages of the infectious cascade may allow more appropriate treatment strategies to be initiated upon initial suspicion of infection, thus minimising the risk of patients being either under- or over-treated—both of which may result in undesirable consequences [21]. To this extent, the use of blood biomarkers may provide an easy and rapid source of clinical information with which to inform and help develop clinical strategies. Of these biomarkers, PCT has been studied extensively in both the ED and ICU [12–15, 22–24], with routine use in the ICU especially widespread. Conversely, novel biomarkers such as MR-proADM remain poorly understood, despite a number of recent studies [16, 18, 19, 25]. Furthermore, biomarker performance may vary extensively depending on whether the infection had developed in the community or during hospitalisation. Hence a key aim of this study focused on the comparison of biomarker and clinical score performance according to the location of initial infection development, following activation of an in-hospital sepsis code.

Based on recent publications, the use of MR-proADM was of especial interest within our investigation, with previous studies showing elevated concentrations resulting from increased levels of microcirculatory and endothelial damage, resulting in the early stages of subsequent organ dysfunction [26–29]. The use of a biomarker to identify developing microcirculatory damage may contribute significant information following the activation of a local, in-hospital sepsis code.

Accordingly, results from this investigation potentially indicate two clinically important uses of MR-proADM following sepsis code activation. Firstly, MR-proADM may be used as a tool to aid in the early identification of disease severity, irrespective of medical setting or prior surgical history. Secondly, it may also be used to aid clinical decision-making in assessing ICU requirement within the ED or on a medical ward.

This study found that MR-proADM had a high accuracy in identifying both 28-day and 90-day mortality, compared to all other biomarkers and clinical scores, although many differences were not found to be significant. Performance was maintained irrespective of whether the infection was developed in the community, culminating in a visit to the ED, or following a surgical procedure. Conversely, the SOFA score appeared to more accurately predict mortality in patients following a hospital-acquired infection, although similarly, differences were not found to be significant. In each case, however, the small sample size and relatively low event rate result in few significant differences between biomarkers and scores, as highlighted when comparing 95% confidence intervals. Nevertheless, similar findings have been shown elsewhere. Elke et al. [25] previously found differences in performance depending on whether the infecting agent consisted solely of Gram positive vs. negative bacteria, as well as highlighting the influence of prior surgical procedures in patients directly admitted onto the ICU. A further ICU study by Andaluz-Ojeda et al. [2] in 326 patients with severe sepsis or septic shock, as well as that of Enguix-Armada [30] in 388 patients with septic shock, also found MR-proADM to have the highest predictive value compared to conventional biomarkers such as PCT, CRP and lactate, although interestingly, the greatest performance was found in patients with a lower degree of organ failure. Nevertheless, no subgroup analysis was performed in either study relating to location of infection development or prior surgical history. It is, however, interesting to note that similar results to these high severity, critical care investigations could be observed in our less severe cohort across three different hospital departments. Accordingly, a significant body of evidence now appears to have been formed across all hospital departments, highlighting the use of MR-proADM in identifying the patients at risk of mid- and long-term mortality.

In contrast, few studies have assessed MR-proADM performance in identifying patients requiring ICU admission. An early recognition of such patients is crucial, since it has previously been shown that inappropriate ED discharge can result in higher mortality rates in patients initially classified as suitable for outpatient treatment, but later directly admitted onto the ICU. Similar findings were also reported after initial triage onto a medical ward [31]. The relatively high performance of MR-proADM in identifying these patients after presentation to the ED, with a modest performance during treatment on a medical ward, may provide clinicians with an additional tool in assessing suitability for ICU admission. Nevertheless, further observational and interventional studies are required to confirm these initial findings, and determine the actual clinical value compared to standard routine practice.

We note several limitations and strengths of this study that deserve greater discussion. This was a single-centre pilot study with a relatively low number of enrolled patients, resulting in many subgroups being underpowered for their respective endpoints. Results must therefore be treated with caution. Nevertheless, similar results were found in comparison to those previously discussed in the literature, despite the patient population enrolled in this study being preselected on the basis of fulfilling an in-house sepsis criteria, and can therefore be viewed as additional collaborative evidence. Similarly, the number of patients where the in-hospital sepsis code was activated, yet no infection was subsequently found, only amounted to 18 (12.2%) patients. A greater degree of confidence in the discriminatory results between infected and non-infected patients could be achieved with a larger sample size. Finally, biomarker incorporation within a sepsis code requires a short turnaround time to result, which may be achieved using point-of-care (POC) technology. At this point in time, however, no such platform exists for the measurement of MR-proADM, making routine incorporation of this biomarker in a clinical setting problematic.

## Conclusions

This study found that the use of PCT to discriminate between infected and non-infected cases following activation of an in-hospital sepsis code, followed by measurement of MR-proADM to determine infection severity and assess the requirement for ICU admission, may help guide initial treatment decisions and intensify therapy in high-risk patients. Interventional studies, specifically focussing on MR-proADM, to potentially optimise early patient treatment strategies are now crucial before implementation in routine clinical use.

## Data Availability

The datasets used and/or analysed during the present study are available from the corresponding author upon reasonable request.

## Abbreviations

*AUROC*: area under the receiver operating characteristic curve
*CI*: confidence interval
*CRB*-*65*: severity scores for community-acquired pneumonia
*CRP*: C-reactive protein
*ED*: emergency department
*HR*: hazard ratio
*IQR*: interquartile range
*MR*-*proADM*: mid-regional proadrenomedullin
*N*: number
*OR*: odds ratio
*PCT*: procalcitonin
*qSOFA*: quick Sequential Organ Failure Assessment
*RRT*: renal replacement therapy
*SIRS*: Systemic Inflammatory Response Syndrome
*SOFA*: Sequential Organ Failure Assessment

## References

[1] Elke G, Bloos F, Wilson DC, Brunkhorst FM, Briegel J, Reinhart K (2018). The use of mid-regional proadrenomedullin to identify disease severity and treatment response to sepsis—a secondary analysis of a large randomised controlled trial. Crit Care.

[2] Andaluz-Ojeda D, Nguyen HB, Meunier-Beillard N (2017). Superior accuracy of mid-regional proadrenomedullin for mortality prediction in sepsis with varying levels of illness severity. Ann Intensive Care..

[3] Levy MM, Fink MP, Marshall JC, Abraham E, Angus D, Cook D (2003). 2001 SCCM/ESICM/ACCP/ATS/SIS international sepsis definitions conference. Crit Care Med.

[4] Singer M, Deutschman CS, Seymour CW (2016). The third international consensus definitions for sepsis and septic shock (Sepsis-3). JAMA..

[5] Seymour CW, Liu VX, Iwashyna TJ, Brunkhorst FM, Rea TD, Scherag A (2016). Assessment of clinical criteria for sepsis: for the third international consensus definitions for sepsis and septic shock (Sepsis-3). JAMA..

[6] Singer AJ, Ng J, Thode HC, Spiegel R, Weingart S (2017). Quick SOFA scores predict mortality in adult emergency department patients with and without suspected infection. Ann Emerg Med.

[7] Simpson SQ (2016). New sepsis criteria: a change we should not make. Chest.

[8] Simpson SQ (2017). SIRS in the time of Sepsis-3. Chest..

[9] Simpson SQ (2017). Diagnosing sepsis: a step forward, and possibly a step back. Ann Transl Med..

[10] Charles PE, Peju E, Dantec A, Bruyere R, Meunier-Beillard N, Dargent A (2017). Mr-Proadm elevation upon ICU admission predicts the outcome of septic patients and is correlated with upcoming fluid overload. Shock..

[11] Schuetz P, Affolter B, Hunziker S, Winterhalder C, Fischer M, Balestra GM (2010). Serum procalcitonin, C-reactive protein and white blood cell levels following hypothermia after cardiac arrest: a retrospective cohort study. Eur J Clin Invest.

[12] Schuetz P, Albrich W, Christ-Crain M, Chastre J, Mueller B (2010). Procalcitonin for guidance of antibiotic therapy. Expert Rev Anti Infect Ther..

[13] Schuetz P, Aujesky D, Muller C, Muller B (2015). Biomarker-guided personalised emergency medicine for all—hope for another hype?. Swiss Med Wkly..

[14] Schuetz P, Christ-Crain M, Muller B (2009). Procalcitonin and other biomarkers to improve assessment and antibiotic stewardship in infections–hope for hype?. Swiss Med Wkly..

[15] Schuetz P, Christ-Crain M, Thomann R, Falconnier C, Wolbers M, Widmer I (2009). Effect of procalcitonin-based guidelines vs standard guidelines on antibiotic use in lower respiratory tract infections: the ProHOSP randomized controlled trial. JAMA.

[16] Saeed K, Wilson DC, Bloos F, Schuetz P, van der Does Y, Melander O (2019). The early identification of disease progression in patients with suspected infection presenting to the emergency department: a multi-centre derivation and validation study. Crit Care.

[17] Gille J, Ostermann H, Dragu A, Sablotzki A (2017). MR-proADM: a new biomarker for early diagnosis of sepsis in burned patients. J Burn Care Res..

[18] Nierhaus A, Bloos F, Wilson DC, Elke G, Meybohm P, SepNet Critical Care Trials G (2018). Predicting the requirement for renal replacement therapy in intensive care patients with sepsis. Crit Care.

[19] Elke G, Bloos F, Wilson DC, Meybohm P (2018). Identification of developing multiple organ failure in sepsis patients with low or moderate SOFA scores. Crit Care.

[20] Ferrer R, Ruiz-Rodriguez JC, Larrosa N, Llaneras J, Molas E, González-López JJ (2017). Sepsis code implementation at Vall d’Hebron university hospital: rapid diagnostics key to success. ICU Manag Pract..

[21] Van der Does Y, Limper M, Jie KE, Schuit SCE, Jansen H, Pernot N (2018). Procalcitonin-guided antibiotic therapy in patients with fever in a general emergency department population: a multicenter noninferiority randomized clinical trial (HiTEMP study). Clin Microbiol Infect..

[22] Schuetz P, Batschwaroff M, Dusemund F, Albrich W, Burgi U, Maurer M (2010). Effectiveness of a procalcitonin algorithm to guide antibiotic therapy in respiratory tract infections outside of study conditions: a post-study survey. Eur J Clin Microbiol Infect Dis.

[23] Schuetz P, Chiappa V, Briel M, Greenwald JL (2011). Procalcitonin algorithms for antibiotic therapy decisions: a systematic review of randomized controlled trials and recommendations for clinical algorithms. Arch Intern Med.

[24] Schuetz P, Wirz Y, Sager R, Christ-Crain M, Stolz D, Tamm M (2017). Procalcitonin to initiate or discontinue antibiotics in acute respiratory tract infections. Cochrane Database Syst Rev.

[25] Elke G, Bloos F, Wilson DC, Brunkhorst FM, Briegel J, Reinhart K (2018). The use of mid-regional proadrenomedullin to identify disease severity and treatment response to sepsis—a secondary analysis of a large randomised controlled trial. Crit Care..

[26] Temmesfeld-Wollbruck B, Hocke AC, Suttorp N, Hippenstiel S (2007). Adrenomedullin and endothelial barrier function. Thromb Haemost.

[27] Pittard AJ, Hawkins WJ, Webster NR (1994). The role of the microcirculation in the multi-organ dysfunction syndrome. Clin Intensive Care..

[28] Xie Z, Chen WS, Yin Y, Chan EC, Terai K, Long LM (2018). Adrenomedullin surges are linked to acute episodes of the systemic capillary leak syndrome (Clarkson disease). J Leukoc Biol.

[29] Vigue B, Leblanc PE, Moati F, Pussard E, Foufa H, Rodrigues A (2016). Mid-regional pro-adrenomedullin (MR-proADM), a marker of positive fluid balance in critically ill patients: results of the ENVOL study. Crit Care.

[30] Enguix-Armada A, Escobar-Conesa R, La Torre AG, De La Torre-Prados MV (2016). Usefulness of several biomarkers in the management of septic patients: c-reactive protein, procalcitonin, presepsin and mid-regional pro-adrenomedullin. Clin Chem Lab Med.

[31] Fernando SM, Rochwerg B, Reardon PM, Thavorn K, Seely AJE, Perry JJ (2018). Emergency department disposition decisions and associated mortality and costs in ICU patients with suspected infection. Crit Care.

